# Bacterial expression of human small GTPases induces stochastic predatory responses and motility interference in *Physarum polycephalum*

**DOI:** 10.1371/journal.pone.0359275

**Published:** 2026-09-24

**Authors:** Rui Saiki, Rion Iimura, Makoto Ichimura, Yoshihito Osada, Ken-Ichi Sano

**Affiliations:** 1 Department of Applied Chemistry, Faculty of Fundamental Engineering, Nippon Institute of Technology, Saitama, Japan; 2 Tochigi Prefectural Oyama High School, Oyama, Tochigi, Japan; 3 Department of Innovative System Engineering, Faculty of Engineering, Nippon Institute of Technology, Saitama, Japan; 4 Glycometabolic Biochemistry Laboratory, RIKEN, Saitama, Japan; 5 Department of Sustainable Biological Chemistry, Faculty of Fundamental Engineering, Nippon Institute of Technology, Saitama, Japan; Okayama University, JAPAN

## Abstract

We investigated the behavioral responses of the slime mold *Physarum polycephalum* to *Escherichia coli* expressing human small GTPases to elucidate how internalized proteins affect its actomyosin-based motility machinery. We found that prey bacteria expressing hKRAS WT or hRhoA triggered a deterministic “conflict” behavior, in which *P. polycephalum* failed to complete ingestion despite physical contact. Notably, the oncogenic hKRAS G12C mutation induced a transition to stochastic switching among predation, conflict, and sampling-mediated rejection, whereas hRac1 expression showed a tendency toward diversified behavioral outcomes. To explain this, we propose a working model centered on molecular jamming or local perturbation of intracellular oscillatory dynamics, in which non-prenylated G-domains act as decoys that decouple the contractile machinery from leading-edge actin dynamics. This behavioral stochasticity likely reflects physical noise arising from the specific conformational states and functional heterogeneity of the expressed proteins. Our results suggest that *P. polycephalum* functions as a sensitive biological sensor of the biophysical properties of prey proteins, providing a unique platform for exploring the co-evolutionary arms race and molecular survival strategies between bacteria and amoeboid predators.

## Introduction

The plasmodium of *Physarum polycephalum* is a multinucleated giant amoeboid organism that exhibits dynamic motility driven by the active formation of lamellipodia and filopodia and by rhythmic actomyosin contractions [1–3]. Despite lacking a central nervous system, this organism demonstrates primitive biological intelligence, as evidenced by its ability to solve optimization problems [4–10]. For instance, the plasmodium can find the shortest path in a maze to connect food sources and solve the traveling salesman problem through neurocomputing based on photophobic responses [5,9]. These intelligent behaviours are realized through the chaotic fluctuations of its oscillatory protoplasmic streaming, which allow the organism to search for optimal solutions and spontaneously destabilize stable states to explore alternatives [5,6]. Thus, *P. polycephalum* represents a unique biological system where complex behavioural patterns and decision-making are governed by its intrinsic rhythmic dynamics [6,7,11–13].

As a multinucleate organism, the plasmodium grows to a macroscopic scale through repeated nuclear divisions without cytokinesis, resulting in a single cell that behaves as a single individual [1]. In this organism, morphological deformation is synonymous with behaviour, driven by actin cytoskeletal reorganization, acto-myosin cytoskeletal reorganization, and internal pressure generated through rhythmic contraction [3,12]. This unique organization enables the simultaneous analysis of molecular information flow, cytoskeletal remodelling, and macroscopic behavioural outputs. By perturbing these pathways at the molecular level, we aim to elucidate the direct link between cytoskeletal dynamics and biological intelligence. Our approach—integrating micro-level signalling with macro-level behavioural analysis—represents a novel fusion of nanotechnology, biotechnology, and information technology.

A cornerstone of our research is the reconstruction of such biological dynamics through an *in vitro* reconstituted approach [14,15]. We have previously developed a functional actin-based gel cross-linked with polyethylene glycol (PEG), which exhibits sustained autonomous oscillation of its storage modulus with a periodicity of 15–25 min [16]. This underscores that the rhythmic motility and decision-making of *P. polycephalum* emerge from the autonomous, multiscale rheological properties of its internal actin machinery.

Complementing this bottom-up study, we further explore how exogenous molecular interference can directly modulate these behavioural patterns through the cytoskeletal machinery. Although various physical and chemical stimuli have been reported to modulate the plasmodium motility, direct regulation through exogenous molecular interference with cytoskeletal dynamics remains an underexplored frontier. One potential method for such manipulation is feeding-based RNA interference (RNAi), in which double-stranded RNA (dsRNA) expressed in prey *Escherichia coli* is ingested by the plasmodium [17,18]. However, establishing an effective feeding RNAi protocol for *P. polycephalum* has remained challenging because of its acute chemosensory system and selective predatory nature [19,20].

In this study, we initially sought to suppress the expression of *ras1*, a member of the small guanosine triphosphatase (small GTPase) superfamily, involved in cytoskeletal regulation, via feeding of RNAi [21]. Surprisingly, we discovered that the presence of Ras1 protein in prey bacteria, even as a result of leaky expression, triggered a distinct “conflict” behaviour in the plasmodium. In this state, the plasmodium approached the bacterial spot but failed to complete ingestion, exhibiting local wandering and stagnation. This interference was consistently observed with human KRAS, RhoA, and Rac1; notably, the oncogenic KRAS G12C mutation G12C mutation induced stochastic switching among conflict, predation, and sampling-mediated avoidance, whereas Rac1 expression showed a tendency toward diversified behavioral outcomes [22–24]. To interpret this behavioral stochasticity, we propose a working model centered on physical noise and molecular jamming or localized signaling perturbation within the motility apparatus, highlighting how *P. polycephalum* acts as a sensitive biological sensor for the physical and conformational states of intracellular proteins. While the amoeba-bacteria interaction is widely recognized as an “evolutionary training ground” for infection and defence, *P. polycephalum*—with its more complex signalling repertoire than *Dictyostelium discoideum*—offers a unique platform to explore how early eukaryotes navigate the fine line between nutrient acquisition and infectious risk [25–27]. This study proposes a novel intersection between protein biophysics and the behavioural determination of microbial predation.

## Materials and methods

### Organisms and culture conditions

The myxomycete *Physarum polycephalum* strain HU195X200 (kindly provided by Dr. Masashi Aono, Keio University) was used throughout this study. Plasmodia were revived from sclerotia stored at −80 °C on filter paper. Sclerotia were cut into approximately 1 cm² pieces and inoculated onto 1% (w/v) plain agar medium (Eiken Chemical Co., Ltd.). Cultures were maintained at 25 °C in the dark and supplemented with moisturized oatmeal. For genetic manipulations, *Escherichia coli* strains XL1-Blue or DH5α were employed. BL21(DE3) and HT115(DE3) strains were utilized for protein and double-stranded RNA (dsRNA) expression, respectively. *E. coli* cells were cultured in LB medium supplemented with 100 μg/mL carbenicillin at 37 °C.

### Plasmid construction and expression

cDNAs of ras1 and hemagglutinin (HA) were cloned into L4440 and pET-3a/3d vectors. Human KRAS, KRAS G12C, hRhoA, and hRac1 sequences were codon-optimized for *E. coli* expression, synthesized (Eurofins Genomics), and cloned into pET-3a and 3d vectors. Protein and dsRNA expression was induced with 0.5 μM IPTG at the mid-log phase for 3 h at 37 °C. Harvested cells were washed twice with PBS (–). For sotorasib treatment, hKRAS G12C-expressing cells were incubated in PBS (–) containing 5 μM sotorasib (MedChemExpress) for 30 min at 25 °C, followed by two washes to remove residual sotorasib. The final bacterial paste was prepared by complete removal of the supernatant.

### Behavioural observation

Bacterial paste was inoculated onto the centre of a 1% (w/v) agar plate, and a 1 cm² block of *P. polycephalum* plasmodium was placed adjacent to it. Time-lapse imaging was performed at 10 min intervals for 24 h using a digital camera (FinePix XP140; Fujifilm) inside a humidified incubator at 25 °C. Due to the 10 min frame interval, high-frequency oscillatory dynamics (1–2 min period) were not resolved; thus, responses were evaluated based on macroscopic behavioral phenotypes observed over 24 h. Behavioural responses were categorized into three defined phenotypes: Predation (defined by the clear consumption by the end of the 24 h period), Conflict (prolonged contact typically lasting 6-12h without sustained feeding), and Sampling-mediated avoidance (initial contact followed by reversal and retreat). Trials that could not be classified within 24 h were designated as “Indeterminable.” For each experimental condition, at least 12 independent trials were performed to calculate the occurrence rate of each phenotype.

### Protein verification

Harvested *E. coli* cells were denatured in SDS-PAGE sample buffer at 95 °C for 10 min. Proteins were separated on 5–20% gradient gels and transferred to PVDF membranes. Following blocking with PBS-BT, membranes were incubated with primary anti-Ras antibody (1:1,000; #3965, Cell Signalling Technology) and an alkaline phosphatase-conjugated secondary antibody (S3731; Promega). Visualization was performed using SIGMA FAST BCIP/NBT.

### Statistical analysis

The occurrence rate of each phenotype was calculated as the percentage of the total number of experiments for each condition. To evaluate the statistical significance of behavioural shifts between groups (e.g., deterministic vs. stochastic behaviour), frequency distributions were compared using the Chi-square test (or Fisher's exact test, *P* < 0.05 being considered statistically significant). All calculations and visualizations were performed using Microsoft Excel, Adobe Photoshop and Illustrator.

## Results

### Ras1 protein expression in *E. coli* induces predatory interference

**Fig 1:**
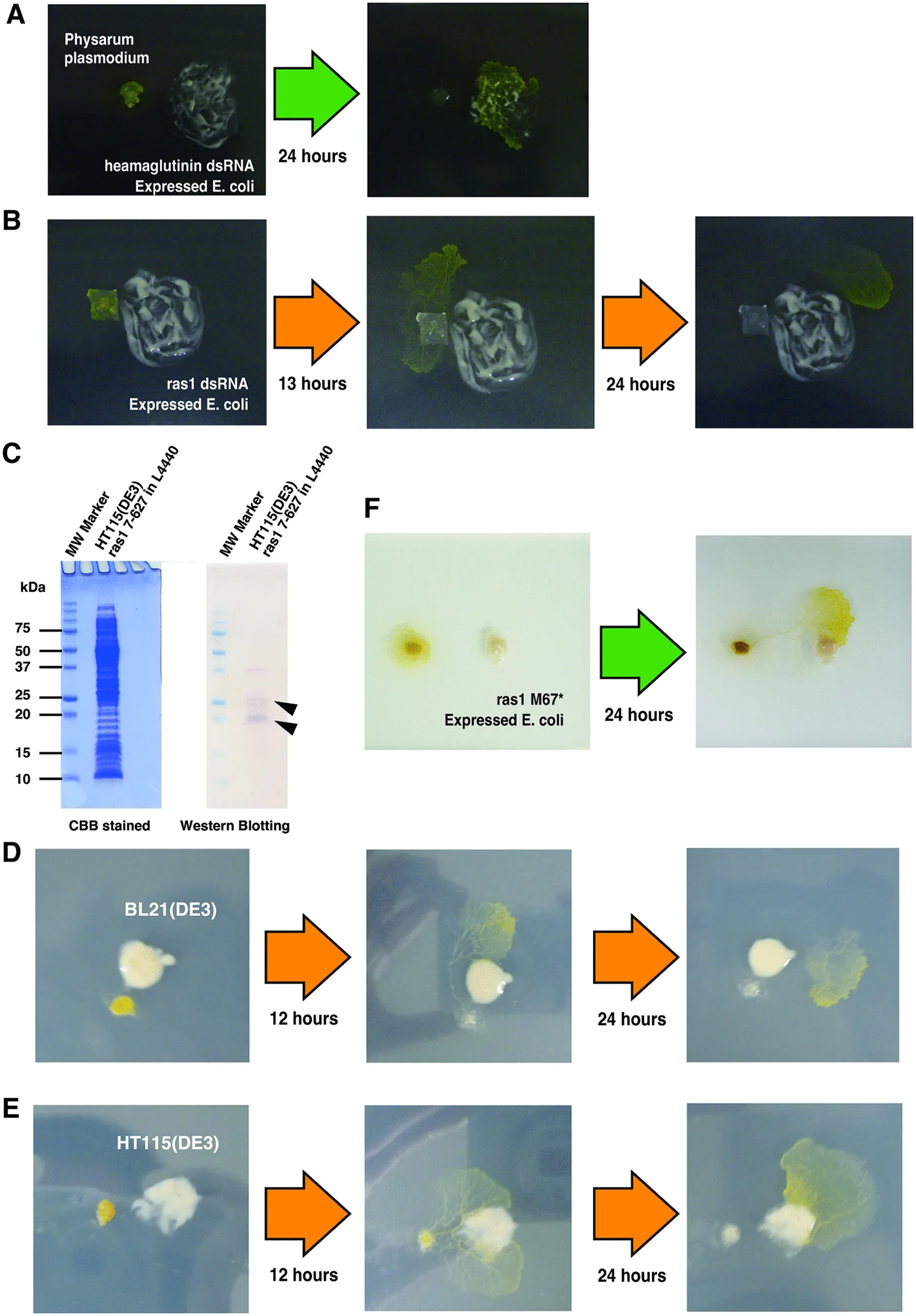
Ras1 protein, but not dsRNA, induces predatory interference in *P. polycephalum.* (A) Normal predatory behaviour. *P. polycephalum* plasmodium completely ingests control *E. coli* cells expressing hemagglutinin (HA) dsRNA. (B) Predatory interference (conflict behaviour) when provided with *E. coli* targeting the endogenous *ras1* gene via dsRNA expression. (C) Detection of leaky Ras1 protein expression. CBB-stained SDS-PAGE gel (left) and western blot analysis (right) of HT115(DE3) cells carrying the *ras1* (residues 7–627) L4440 plasmid. Arrowheads indicate the leaky expression of recombinant Ras1 protein. (D, E) Robustness of the conflict behaviour. The conflict response was consistently observed in both (D) BL21(DE3) and (E) HT115(DE3) strains intentionally overexpressing the Ras1 protein, confirming that the protein itself, rather than the dsRNA, triggers the interference. (F) Rescue of predatory behaviour via elimination of Ras1 protein. *E. coli* expressing a *ras1* variant with a nonsense mutation at M67 (M67*).

We initially attempted to establish a feeding RNAi protocol for *P. polycephalum* using *E. coli* expressing dsRNA targeting the endogenous *ras1* gene. However, the plasmodium exhibited an unexpected “conflict” behaviour toward the *ras1* dsRNA-expressing strain, in which it is willing to eat but unable to ingest the bacteria. While the plasmodium approached the bacterial colony, it failed to complete ingestion, manifesting as localized wandering or stagnation (Fig 1B). This response stood in sharp contrast to the typical predatory expansion observed on control bacteria (Fig 1A). Western blotting revealed leaky expression of Ras1 protein in the dsRNA-expressing strain (Fig 1C). To verify whether the protein itself caused this interference, we overexpressed Ras1 protein in both BL21(DE3) and HT115(DE3) strains. Both strains consistently elicited the same conflict response (Figs 1D & E). Furthermore, the elimination of Ras1 protein expression via a nonsense mutation (M67*) completely rescued the predatory behaviour, allowing the plasmodium to successfully consume the bacteria within 24 h (Fig 1F). These results indicate that the presence of the Ras1 protein, rather than the dsRNA, induces predatory interference.

### Behavioural responses to human KRAS

**Fig 2:**
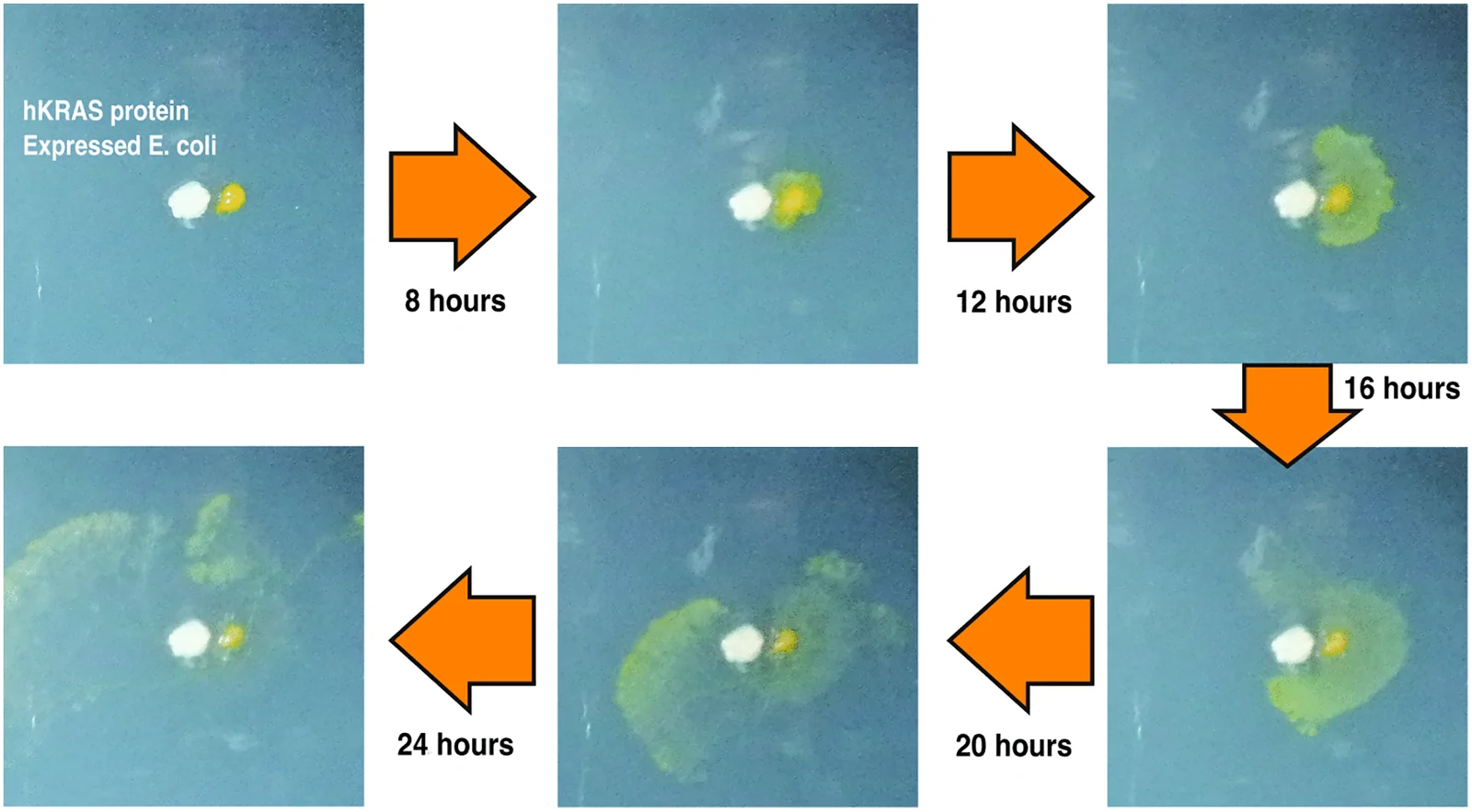
Consistent conflict behaviour of *P. polycephalum* towards *E. coli* expressing human KRAS (WT). Time-lapse images showing the behavioural response of *P. polycephalum* over 24 h.

**Fig 3:**
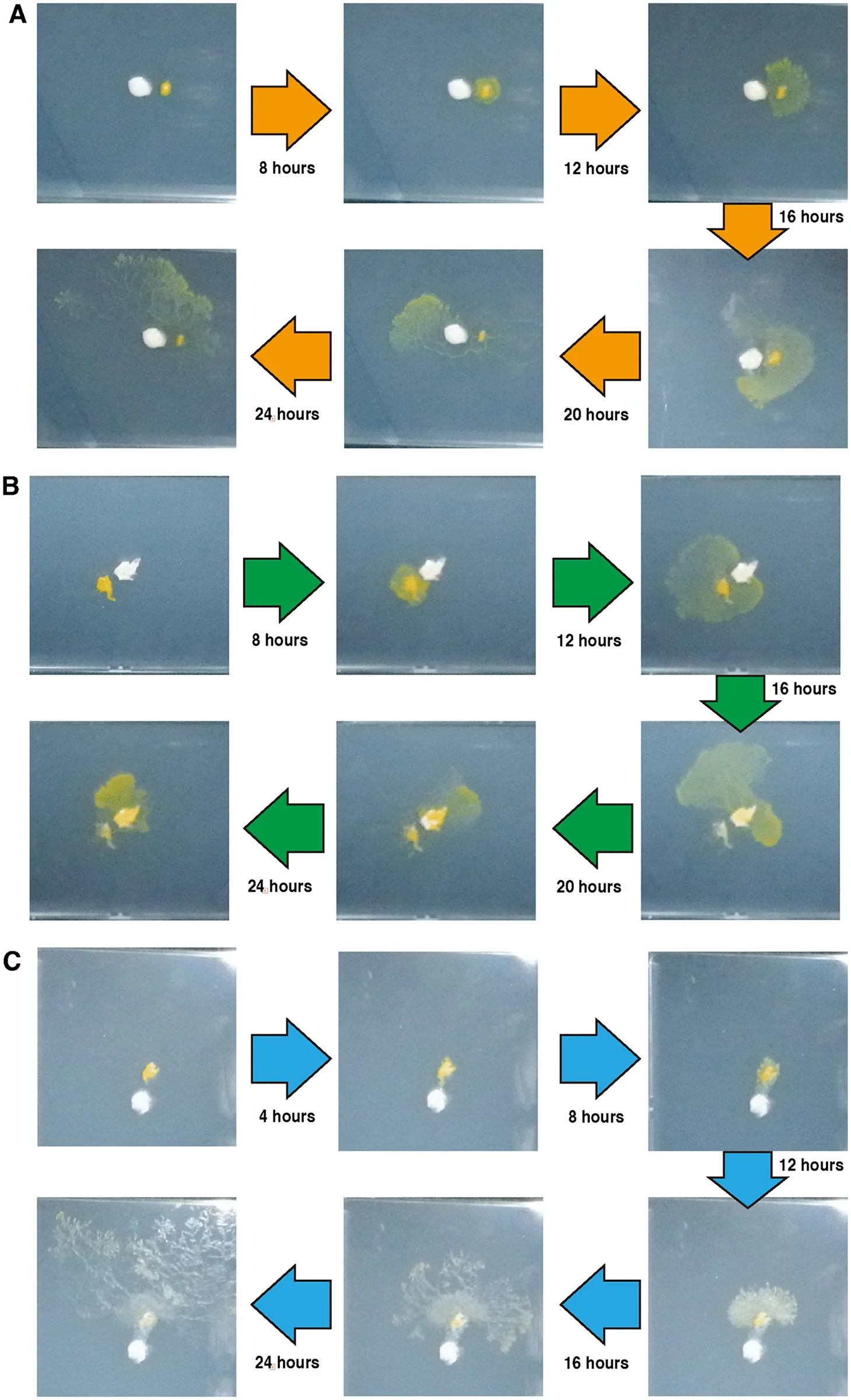
Stochastic behavioural outcomes of *P. polycephalum* towards *E. coli* expressing oncogenic human KRAS G12C. Time-lapse images illustrating three distinct behavioural patterns induced by the hKRAS G12C mutant. (A) Conflict behaviour, (B) predation, and (C) sampling-mediated avoidance.

To investigate the generality of this phenomenon, we examined behavioural responses to human small GTPases [21,28,29]. Among the various human Ras isoforms, KRAS is of particular clinical importance; notably, molecular targeted drugs have been developed to specifically inhibit the KRAS G12C mutant [23,24].　When provided with *E. coli* expressing wild-type human KRAS (hKRAS WT), the plasmodia exhibited a deterministic conflict behaviour in 100% of trials (12/12, Table 1, Fig 2; S1 Video). In contrast, the introduction of the oncogenic hKRAS G12C mutation shifted this deterministic response to a stochastic one. The behavioural outcomes branched into conflict (12/18, 66.7%), predation (4/18, 22.2%), and sampling-mediated avoidance (2/18, 11.1%) (n = 18, Table 1, Fig 3; S2-S4 Video). Statistical analysis confirmed that the behavioural distribution of the hKRAS G12C group was significantly different from the hKRAS WT group (*p* = 0.025, Fisher’s exact test). Treatment with the covalent inhibitor sotorasib, which targets the Cys12 residue, yielded a behavioral distribution similar to that of the hKRAS G12C group (*p* = 0.731) and remained significantly different from the hKRAS WT group (*p* = 0.009) (n = 18, Table 1) [23].

**Table 1:** Behavioural responses of *P. polycephalum* to *E. coli* expressing human small GTPases.

Strain / Variant	n	Conflict (%)	Predation (%)	Sampling-mediated avoidance (%)	Indeterminable (%)	p-value (vs. WT)
**hKRAS** **WT**	12	100	0	0	0	–
**hKRAS** **G12C**	18	66.7	22.2	11.1	0	0.025 *
**G12C** **+** **Sotorasib**	18	61.1	33.3	5.6	0	0.009 *
**hRhoA**	12	91.7	0	0	8.3	1
**hRac1**	12	75	16.7	8.3	0	**0.232**

Behavioural responses were categorized into three defined phenotypes: “Conflict” (sustained oscillation at the contact site), “Predation” (complete engulfment and ingestion), and “Sampling-mediated avoidance” (localized contact followed by definitive retreat), with unclassifiable cases designated as “Indeterminable.”

*p*-values were calculated using Fisher’s exact test, comparing the behavioural distribution of each group against the hKRAS WT control group.

* Indicates a significant difference from the hKRAS WT group (*p* < 0.05).

### Divergent responses to Rho-family GTPases

**Fig 4:**
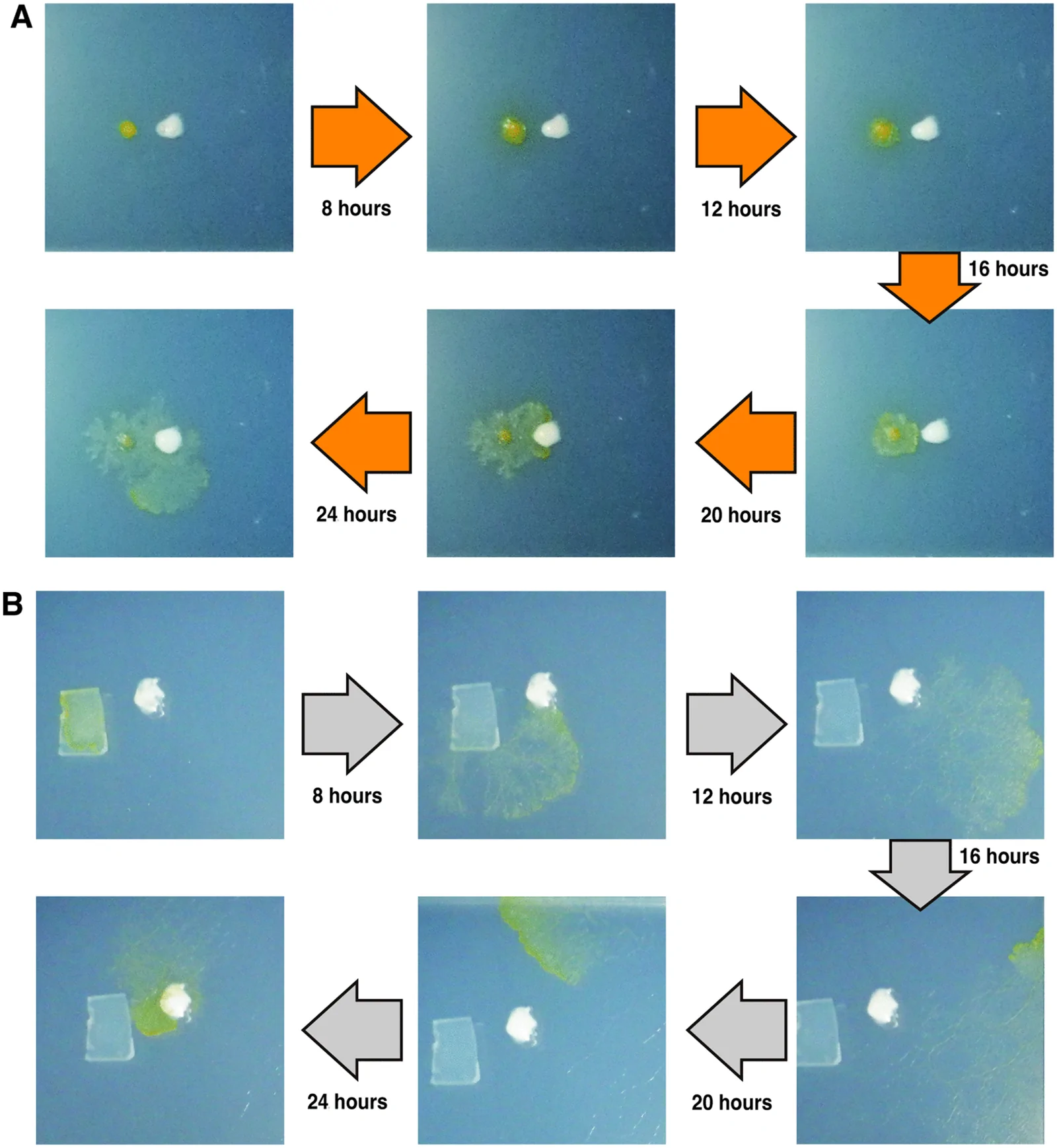
(A) Consistent conflict behaviour of *P. polycephalum* towards *E. coli* expressing human RhoA. Time-lapse images showing the behavioural response of *P. polycephalum* over 24 h. (B) Indeterminable behaviour of *P. polycephalum.* The plasmodium initially approached the *E. coli* (8 h) but subsequently bypassed the colony between 12 and 16 h. It then migrated along the periphery of the Petri dish (20 h) and eventually encircled the *E. coli* by 24 h.

**Fig 5:**
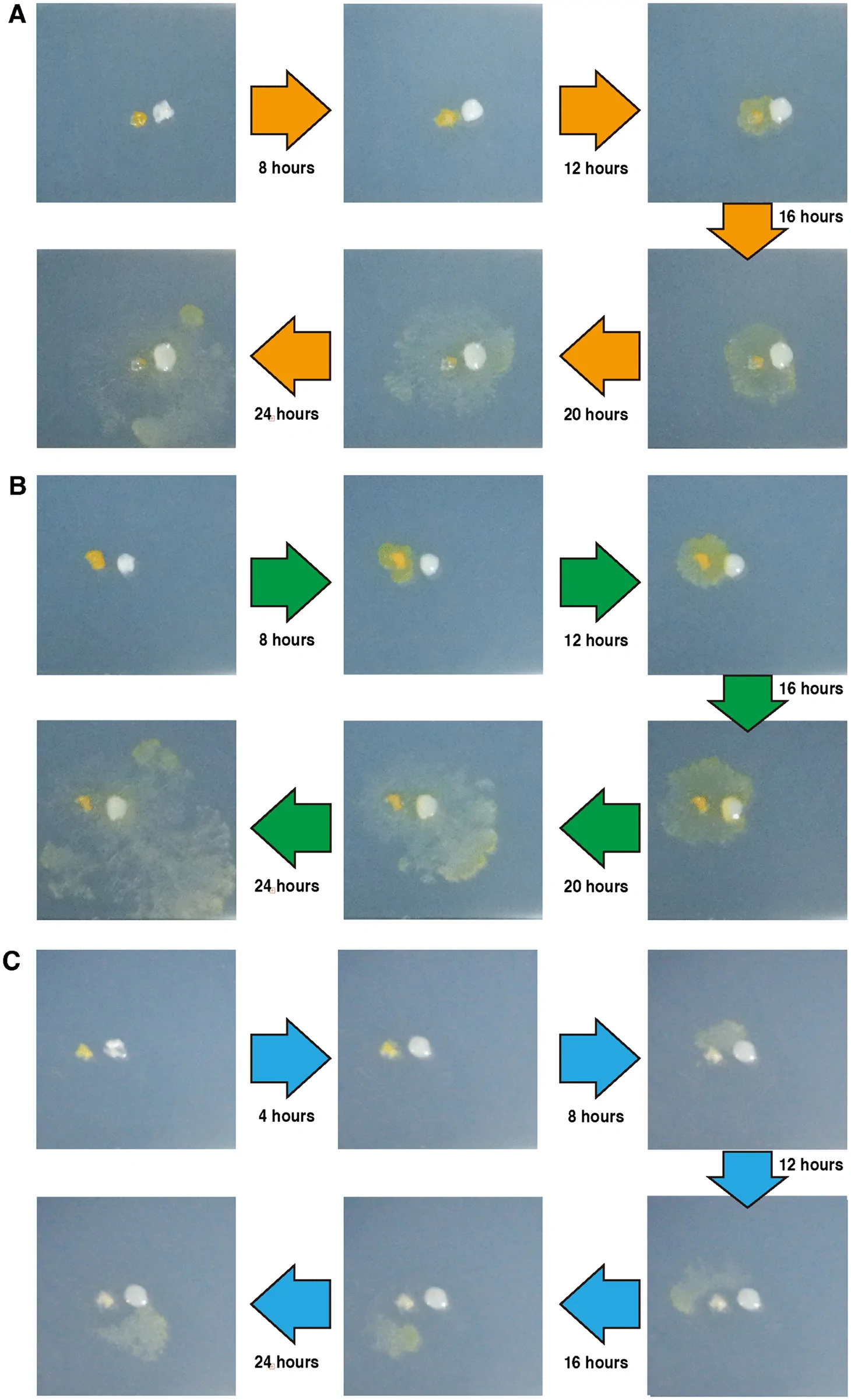
Stochastic behavioural outcomes of *P. polycephalum* towards *E. coli* expressing hRac1. Time-lapse images illustrating three distinct behavioural patterns induced by hRac1. (A) Conflict behaviour, (B) predation, and (C) sampling-mediated avoidance.

We further examined the Rho-family GTPases, RhoA and Rac1, which are key regulators of the actin cytoskeleton [30,31]. Human RhoA (hRhoA) induced a deterministic conflict response in 91.7% (11/12) of cases, similar to hKRAS WT (n = 12, Table 1, Fig 4). However, human Rac1 (hRac1) resulted in a more stochastic distribution, with 75.0% (9/12) conflict, 16.7% (2/12) predation, and 8.3% (1/12) sampling-mediated avoidance behaviours (Table 1, Fig 5). Although a tendency toward diversified behavioral outcomes in the hRac1 group was observed (25.0% total of predation and avoidance), the difference compared to the hKRAS WT group did not reach statistical significance (*p* = 0.232, Fisher’s exact test). In all trials, protoplasmic streaming remained active, suggesting that the interference was localized to the leading edge of the plasmodium.

## Discussion

### Differentiating experimental observations

**Fig 6:**
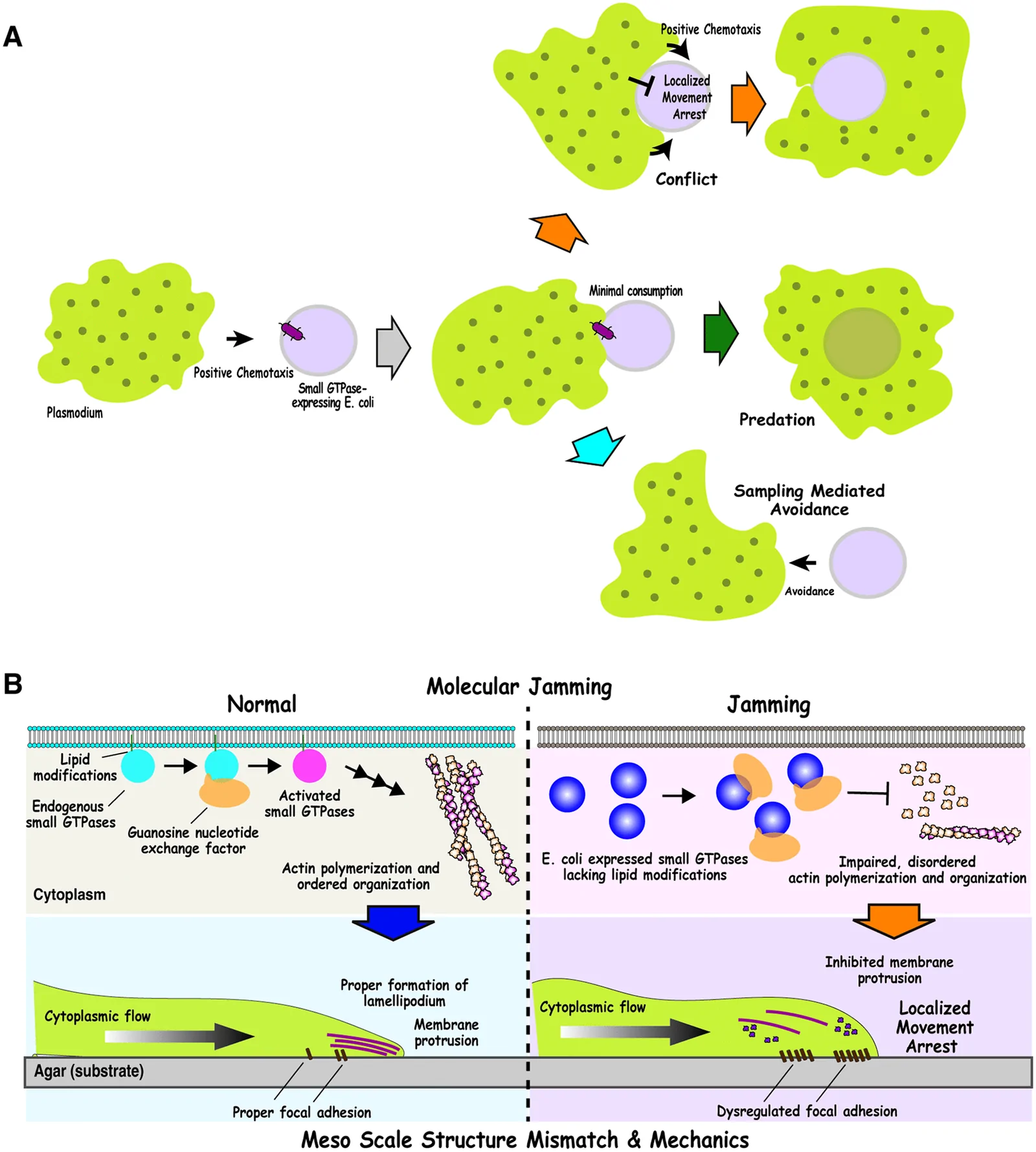
Schematics of behavioral decision-making and proposed mechanistic models. (A) Macroscopic modes of behavioural decision-making in *Physarum polycephalum* during predatory interactions. (B) Working model for localized movement arrest at the leading edge, illustrating potential mechanisms including localized molecular jamming and phase desynchronization of rhythmic contractions.

Fig 6A summarizes the macroscopic modes of behavioural decision making in the *P. polycephalum* plasmodium. Our results demonstrate that the presence of hKRAS WT or hRhoA in prey *E. coli* disrupts the normal predatory behaviour of *P. polycephalum,* shifting it into a deterministic “conflict” state, whereas hKRAS G12C induced stochastic behavioral outcomes, and hRac1 showed a tendency toward diversified outcomes.

Our behavioural observations reveal that *P. polycephalum* initially approaches the *E. coli* colony through positive chemotaxis and initiates predation. However, when encountering bacteria expressing hKRAS WT or hRhoA, the leading edge macroscopically appeared locally arrested specifically at the contact site. While these contact points appeared locally restricted, the rest of the plasmodium—driven by continued chemotactic attraction—attempts to surround the bacterial colony. This spatial mismatch between localized arrest and global attraction leads to a state we termed “conflict”: a sustained behavioural tension in which the organism is willing to eat but unable to ingest, consistent with our proposed model of localized molecular jamming (Fig 6B). This phenomenon suggests that such localized mechanical disruption prevents the coordination required for complete engulfment.

### Integration of molecular jamming and oscillatory phase dynamics

One plausible interpretation is a “molecular jamming” model at the leading edge to explain this interference (Fig 6B). Small GTPases expressed in *E. coli* lack C-terminal lipid modifications (e.g., prenylation), which are essential for membrane anchoring and functional signalling in their native eukaryotic context [32]. When ingested, these non-prenylated G-domains may act as dominant-negative decoys, sequestering endogenous guanosine nucleotide exchange factors GEFs or downstream effectors. This could induce a critical mismatch: while the global contractile engine continues pumping protoplasm forward, the leading edge becomes physically immobilized, forcing internal pressure to redistribute laterally into unproductive localized oscillations [33,34].

Alternatively, these observations can be interpreted through the framework of classical *Physarum* oscillatory dynamics. Pioneering work by Wohlfarth-Bottermann, Matsumoto and co-workers, and Ueda and co-workers established that local perturbations of contraction rhythm, phase wave propagation, and shuttle streaming velocity can directly alter directional migration and taxis without requiring complete mechanical arrest [35–37]. In the present study, parameters such as local contraction period, phase synchronization, and shuttle streaming velocity were not directly measured. Therefore, an equally compelling explanation is that internalized GTPases locally perturb intracellular signaling, resulting in phase desynchronization of rhythmic contractions at the contact zone. Crucially, molecular jamming and oscillatory phase desynchronization are not mutually exclusive; initial molecular jamming at the leading edge could perturb local signal integration, which subsequently disrupts oscillatory phase synchronization and manifests as the macroscopically observed conflict behavior.

### Determinism vs. stochasticity: The role of protein aggregation

A key finding of this study is the shift from deterministic to stochastic behaviour induced by the hKRAS G12C mutation. This stochasticity of hKRAS G12C likely reflects the physical heterogeneity of the interfering proteins within the bacterial cells. The Cys12 residue of the hKRAS G12C mutant is highly reactive and prone to forming intermolecular disulfide-bonded aggregates during protein overexpression in the bacterial cytoplasm, which we hypothesize introduces “physical noise” into the leading-edge machinery. Consistent with this view, sotorasib treatment—which targets the specific -SH group of the G12C mutation—which specifically blocks Cys12—did not alter the stochastic distribution [23,24]. Rather than executing a distinct biochemical pathway, we propose as a working model that the non-uniform distribution of G12C aggregates may destabilize the local leading-edge dynamics, allowing intrinsic oscillatory fluctuations to tip decision-making toward either predation or retreat. It should be noted, however, that protein aggregation and soluble/insoluble fractions were not directly measured in this study, and this mechanism remains a proposed explanation.

### Mechanistic insight from Rho-family signalling at the leading edge

The contrast between hRhoA and hRac1 provides additional insight into the spatial regulation of plasmodial motility. RhoA typically governs actomyosin contractility and the maturation of focal adhesions, and its interference likely induces a “molecular anchoring” effect that rigidly fixes the leading edge. In contrast, Rac1 is the primary orchestrator of membrane protrusion, coordinating dynamic actin polymerization at the lamellipodia [38,39]. While the behavioural shift for hRac1 did not reach statistical significance in this study, the observed stochasticity may stem from its distinct regulatory network. Exogenous, non-prenylated Rac1 is hypothesized to act as a decoy for Rac1-specific GEFs or GAPs that are spatio-temporally distinct from those targeting KRAS or RhoA [29,31,39,40]. Given Rac1's central role in driving the leading-edge protrusion, such interference might destabilize the steering of the plasmodial front rather than imposing a complete arrest. This “unstable steering” could lead to the observed behavioural bifurcations, where local fluctuations in the leading-edge dynamics may help determine whether the organism proceeds with predation or retreats.

### Deciphering microbial warfare: The molecular logic of predatory decision-making

The ability of *P. polycephalum* to discriminate between different protein states carries significant evolutionary implications. The long-standing predator-prey relationship between amoebae and bacteria serves as an “evolutionary training ground” for both bacterial infection strategies and eukaryotic defence mechanisms [25,26]. Although these pathogens primarily utilize these effectors to invade host cells, early amoeboid predators must have faced strong evolutionary pressure to detect and counter such molecular mimicry during ingestion. Pathogenic bacteria, such as *Salmonella*, utilize specialised effectors that mimic or modulate host signalling molecules to evade digestion [27,41,42]. For instance, *Salmonella* delivers effectors like SopE (a GEF mimic) and SptP (a GAP mimic) to directly manipulate host Rho-family GTPases, thereby controlling actin dynamics and membrane trafficking to facilitate its survival within the host [27,41,42].

Our results raise the possibility that *P. polycephalum* has evolved to sense such molecular perturbations with high sensitivity. In this context, *P. polycephalum* may offer a superior experimental platform compared to the widely used model organism *Dictyostelium discoideum.* While *D. discoideum* possesses a streamlined and well-characterised signalling system, its repertoire of sensory receptors and signal integration pathways is relatively limited. In contrast, the *P. polycephalum* genome reveals a vastly expanded array of G-protein-coupled receptors (GPCRs) and ancestral two-component systems, reflecting a more complex evolutionary history of environmental sensing [43]. This increased molecular complexity likely functions as a high-resolution biological sensor, enabling the plasmodium to integrate subtle conformational and physical perturbations. To directly test the proposed hypotheses, future studies incorporating direct optical measurements of local contraction rhythms, soluble/insoluble protein fractionations, and quantitative motility tracking will be valuable.

Further investigating the specific behavioural outputs triggered by these bacterial-mimetic proteins, will provide new insights into the co-evolutionary arms race and the strategic competition between bacteria and amoeboid predators. Such investigations will clarify how early eukaryotes navigate the fine line between nutrient acquisition and infectious risk, positioning *P. polycephalum* as a powerful organismal platform for decoding the molecular logic of microbial warfare at the interface of protein biophysics and ethology.

## Supporting information

S1 Video Consistent conflict behaviour of *Physarum polycephalum* towards *Escherichia coli* expressing human KRAS (WT). Time-lapse movie showing the behavioural response of *P. polycephalum* over 24 h (frames captured at 10 min intervals).
(MP4)

S2 Video Stochastic conflict behaviour of *Physarum polycephalum* towards *Escherichia coli* expressing oncogenic human KRAS G12C. Time-lapse movie illustrating the first of three distinct behavioural patterns induced by the hKRAS G12C mutant over 24 h (1 frame = 10 min), representing the conflict behaviour.
(MP4)

S3 Video Stochastic predation behaviour of *Physarum polycephalum* towards *Escherichia coli* expressing oncogenic human KRAS G12C. Time-lapse movie illustrating the second of three distinct behavioural patterns induced by the hKRAS G12C mutant over 24 h (1 frame = 10 min), representing the predation behaviour.
(MP4)

S4 Video Stochastic sampling-mediated avoidance behaviour of *Physarum polycephalum* towards *Escherichia coli* expressing oncogenic human KRAS G12C. Time-lapse movie illustrating the third of three distinct behavioural patterns induced by the hKRAS G12C mutant over 24 h (1 frame = 10 min), representing the sampling-mediated avoidance.
(MP4)

S1 Table Raw data of individual behavioral trials. Complete list of independent experimental trials recorded for each bacterial variant, showing the experiment date, sample condition, and classified behavioral phenotype over 24 h of observation.
(XLSX)

## References

[1] Gray WD, Alexopoulos CJ. Biology of the Myxomycetes. New York: Ronald Press Co.; 1968.

[2] Kamiya N. Protoplasmic streaming. Springer-Verlag; 1959.

[3] Kessler D. Plasmodial structure and motility. Cell Biology of Physarum and Didymium. Elsevier; 1982. p. 145–208. doi: 10.1016/b978-0-12-049601-3.50010-9

[4] Aono M, Gunji Y-P. Beyond input-output computings: Error-driven emergence with parallel non-distributed slime mold computer. Biosystems. 2003;71(3):257–87. doi: 10.1016/s0303-2647(03)00085-6 14563567

[5] Aono M, Hara M. Spontaneous deadlock breaking on amoeba-based neurocomputer. Biosystems. 2008;91(1):83–93. doi: 10.1016/j.biosystems.2007.08.004 17889990

[6] Aono M, Hirata Y, Hara M, Aihara K. Amoeba-based chaotic neurocomputing: combinatorial optimization by coupled biological oscillators. New Generation Computing. 2009;27(2):129–57. doi: 10.1007/s00354-008-0058-4

[7] Aono M, Naruse M, Kim S-J, Wakabayashi M, Hori H, Ohtsu M, et al. Amoeba-inspired nanoarchitectonic computing: solving intractable computational problems using nanoscale photoexcitation transfer dynamics. Langmuir. 2013;29(24):7557–64. doi: 10.1021/la400301p 23565603

[8] Nakagaki T, Guy RD. Intelligent behaviors of amoeboid movement based on complex dynamics of soft matter. Soft Matter. 2007;4(1):57–67. doi: 10.1039/b706317m 32907084

[9] Nakagaki T, Yamada H, Tóth A. Maze-solving by an amoeboid organism. Nature. 2000;407(6803):470. doi: 10.1038/35035159 11028990

[10] Tero A, Takagi S, Saigusa T, Ito K, Bebber DP, Fricker MD, et al. Rules for biologically inspired adaptive network design. Science. 2010;327(5964):439–42. doi: 10.1126/science.1177894 20093467

[11] Kobayashi R, Tero A, Nakagaki T. Mathematical model for rhythmic protoplasmic movement in the true slime mold. J Math Biol. 2006;53(2):273–86. doi: 10.1007/s00285-006-0007-0 16770610

[12] Nakagaki T, Ueda T. Phase switching of oscillatory contraction in relation to the regulation of amoeboid behavior by the plasmodium of Physarum polycephalum. J Theor Biol. 1996;179(3):261–7. doi: 10.1006/jtbi.1996.0065 8762336

[13] Tero A, Kobayashi R, Nakagaki T. A mathematical model for adaptive transport network in path finding by true slime mold. J Theor Biol. 2007;244(4):553–64. doi: 10.1016/j.jtbi.2006.07.015 17069858

[14] Sano K-I, Kawamura R, Tominaga T, Nakagawa H, Oda N, Ijiro K, et al. Thermoresponsive microtubule hydrogel with high hierarchical structure. Biomacromolecules. 2011;12(5):1409–13. doi: 10.1021/bm101578x 21428377

[15] Sano K-I, Kawamura R, Tominaga T, Oda N, Ijiro K, Osada Y. Self-repairing filamentous actin hydrogel with hierarchical structure. Biomacromolecules. 2011;12(12):4173–7. doi: 10.1021/bm2009922 22011361

[16] Sano K-I, Kawamura R, Osada Y. Autonomous chemo-mechanical oscillations in crosslinked filamentous actin gels. Soft Matter. 2026;22(14):2623–8. doi: 10.1039/d5sm01205h 41810617

[17] Hannon GJ. RNA interference. Nature. 2002;418(6894):244–51. doi: 10.1038/418244a 12110901

[18] Timmons L, Fire A. Specific interference by ingested dsRNA. Nature. 1998;395(6705):854. doi: 10.1038/27579 9804418

[19] Jacobson DN. Locomotion of Physarum polycephalum amoebae is guided by a short range interaction with *E. coli*. Exp Cell Res. 1980;125(2):441–52. doi: 10.1016/0014-4827(80)90138-x 6986276

[20] Saddler JN, Coote JG, Wardlaw AC. Degradation of bacterial lipopolysaccharide by the slime mould Physarum polycephalum. Can J Microbiol. 1979;25(2):124–9. doi: 10.1139/m79-020 373869

[21] Simanshu DK, Nissley DV, McCormick F. RAS proteins and their regulators in human disease. Cell. 2017;170(1):17–33. doi: 10.1016/j.cell.2017.06.009 28666118 PMC5555610

[22] Canon J, Rex K, Saiki AY, Mohr C, Cooke K, Bagal D, et al. The clinical KRAS(G12C) inhibitor AMG 510 drives anti-tumour immunity. Nature. 2019;575(7781):217–23. doi: 10.1038/s41586-019-1694-1 31666701

[23] Hong DS, Fakih MG, Strickler JH, Desai J, Durm GA, Shapiro GI, et al. KRASG12C Inhibition with sotorasib in advanced solid tumors. N Engl J Med. 2020;383(13):1207–17. doi: 10.1056/NEJMoa1917239 32955176 PMC7571518

[24] Huang L, Guo Z, Wang F, Fu L. KRAS mutation: From undruggable to druggable in cancer. Signal Transduct Target Ther. 2021;6(1):386. doi: 10.1038/s41392-021-00780-4 34776511 PMC8591115

[25] Molmeret M, Horn M, Wagner M, Santic M, Abu Kwaik Y. Amoebae as training grounds for intracellular bacterial pathogens. Appl Environ Microbiol. 2005;71(1):20–8. doi: 10.1128/AEM.71.1.20-28.2005 15640165 PMC544274

[26] Butler RE, Smith AA, Mendum TA, Chandran A, Wu H, Lefrançois L, et al. Mycobacterium bovis uses the ESX-1 Type VII secretion system to escape predation by the soil-dwelling amoeba Dictyostelium discoideum. ISME J. 2020;14(4):919–30. doi: 10.1038/s41396-019-0572-z 31896783 PMC7082363

[27] Zhao H, Zhang X, Zhang N, Zhu L, Lian H. The interplay between Salmonella and host: Mechanisms and strategies for bacterial survival. Cell Insight. 2025;4(2):100237. doi: 10.1016/j.cellin.2025.100237 40177681 PMC11964643

[28] Barbacid M. Ras genes. Annu Rev Biochem. 1987;56:779–827. doi: 10.1146/annurev.bi.56.070187.004023 3304147

[29] Song S, Cong W, Zhou S, Shi Y, Dai W, Zhang H, et al. Small GTPases: Structure, biological function and its interaction with nanoparticles. Asian J Pharm Sci. 2019;14(1):30–9. doi: 10.1016/j.ajps.2018.06.004 32104436 PMC7032109

[30] Hall A. Rho GTPases and the actin cytoskeleton. Science. 1998;279(5350):509–14. doi: 10.1126/science.279.5350.509 9438836

[31] Mosaddeghzadeh N, Ahmadian MR. The RHO Family GTPases: Mechanisms of regulation and signaling. Cells. 2021;10(7):1831. doi: 10.3390/cells10071831 34359999 PMC8305018

[32] Hancock JF, Magee AI, Childs JE, Marshall CJ. All ras proteins are polyisoprenylated but only some are palmitoylated. Cell. 1989;57(7):1167–77. doi: 10.1016/0092-8674(89)90054-8 2661017

[33] Paterson HF, Self AJ, Garrett MD, Just I, Aktories K, Hall A. Microinjection of recombinant p21rho induces rapid changes in cell morphology. J Cell Biol. 1990;111(3):1001–7. doi: 10.1083/jcb.111.3.1001 2118140 PMC2116288

[34] Ridley AJ, Hall A. The small GTP-binding protein rho regulates the assembly of focal adhesions and actin stress fibers in response to growth factors. Cell. 1992;70(3):389–99. doi: 10.1016/0092-8674(92)90163-7 1643657

[35] Wohlfarth-Bottermann KE. Oscillatory contraction activity in Physarum. J Exp Biol. 1979;81:15–32. doi: 10.1242/jeb.81.1.15 512576

[36] Ueda T, Mori Y, Kobatake Y. Patterns in the distribution of intracellular ATP concentration in relation to coordination of amoeboid cell behavior in Physarum polycephalum. Exp Cell Res. 1987;169(1):191–201. doi: 10.1016/0014-4827(87)90237-0 3817013

[37] Matsumoto K, Ueda T, Kobatake Y. Reversal of thermotaxis with oscillatory stimulation in the plasmodium of Physarum polycephalum. J Theor Biol. 1988;131(2):175–82. doi: 10.1016/S0022-5193(88)80235-2

[38] Ridley AJ, Paterson HF, Johnston CL, Diekmann D, Hall A. The small GTP-binding protein rac regulates growth factor-induced membrane ruffling. Cell. 1992;70(3):401–10. doi: 10.1016/0092-8674(92)90164-8 1643658

[39] Nobes CD, Hall A. Rho, rac, and cdc42 GTPases regulate the assembly of multimolecular focal complexes associated with actin stress fibers, lamellipodia, and filopodia. Cell. 1995;81(1):53–62. doi: 10.1016/0092-8674(95)90370-4 7536630

[40] Smith KR, Rajgor D, Hanley JG. Differential regulation of the Rac1 GTPase-activating protein (GAP) BCR during oxygen/glucose deprivation in hippocampal and cortical neurons. J Biol Chem. 2017;292(49):20173–83. doi: 10.1074/jbc.M117.796292 29046349 PMC5724004

[41] Davidson A, Hume PJ, Greene NP, Koronakis V. Salmonella invasion of a cell is self-limiting due to effector-driven activation of N-WASP. iScience. 2023;26(5):106643. doi: 10.1016/j.isci.2023.106643 PMC10164908 37168569

[42] Galán JE. Salmonella Typhimurium and inflammation: a pathogen-centric affair. Nat Rev Microbiol. 2021;19(11):716–25. doi: 10.1038/s41579-021-00561-4 34012042 PMC9350856

[43] Schaap P, Barrantes I, Minx P, Sasaki N, Anderson RW, Bénard M, et al. The *Physarum polycephalum* genome reveals extensive use of prokaryotic two-component and metazoan-type tyrosine kinase signaling. Genome Biol Evol. 2015;8(1):109–25. doi: 10.1093/gbe/evv237 26615215 PMC4758236

